# Abundance-weighted pathway mapping demonstrates family-level structure of butyrate and propionate production across the human gut microbiome

**DOI:** 10.1093/ismeco/ycag075

**Published:** 2026-03-26

**Authors:** Rebecca Christensen, Yu Han Daisy Wang, Markus Arnoldini, Jonas Cremer

**Affiliations:** Department of Biology, Stanford University, Stanford, California 94305, United States; Department of Microbiology & Immunology, Columbia University Irving Medical Center, New York, NY 10032, United States; Department of Biology, Stanford University, Stanford, California 94305, United States; Department of Bioengineering, Stanford University, Stanford, California 94305, United States; Department of Health Sciences and Technology, ETH Zurich, 8092 Zurich, Switzerland; Department of Biology, Stanford University, Stanford, California 94305, United States; Bio-X, Stanford University, Stanford, California 94305, United States

**Keywords:** butyrate, propionate, gut microbiome, fermentation pathway, metagenomics

## Abstract

Fermentation products released by bacteria in the large intestine, such as butyrate and propionate, play central roles in host physiology and health. While the metabolic pathways producing these short-chain fatty acids (SCFAs) are well-characterized, less is known about their relative prevalence across hosts and gut conditions. Here, we introduce a genome-resolved, abundance-weighted bioinformatics framework that integrates pathway-based gene identification with extensive literature validation to systematically quantify the potential for butyrate and propionate production across bacterial species and human gut microbiomes. By comparing pathway predictions against over 700 experimentally characterized strains, we demonstrate high concordance with reported metabolic phenotypes, validating our approach beyond prior purely computational studies. Weighted by species abundance across ~18 000 metagenomic samples, we find that dominant gut taxa disproportionately drive SCFA production, with butyrate pathways enriched in Bacillota and propionate pathways in Bacteroidota. This abundance-weighted analysis reveals that pathway presence is well conserved at the family level, highlighting the ecological relevance of dominant taxa for community-level fermentation potential. Our results further show pronounced inter-individual variation and associations with age, birthing method, and inflammatory bowel disease, emphasizing how shifts in microbiota composition influence SCFA availability. By combining pathway-level resolution, abundance-weighted inference, and literature-based validation, our framework provides a robust, scalable approach to link microbial functional potential with host-relevant outcomes.

## Introduction

The human gut harbors a dense microbial ecosystem sustained by continual bacterial growth [1, 2]. In the anaerobic large intestine, bacterial growth is primarily fueled by the fermentation of complex carbohydrates, resulting in a substantial exchange of fermentation products between the microbiome and the host [3]. Approximately 80% of the carbon derived from microbially-consumed carbohydrates in the gut is converted into short-chain fatty acids (SCFAs) such as acetate, butyrate, and propionate [4], making these the most exchanged molecules between gut microbiota and their host.

SCFAs influence numerous aspects of host physiology, including immune and inflammatory responses and antitumor activity [5–13], and provide 2%–10% of daily energy depending on diet [4]. Individual SCFAs exert distinct metabolic roles: butyrate fuels colonic epithelial cells [5, 14–16], whereas propionate enters circulation and is largely metabolized in the liver [14, 15, 17]. A reduction in butyrate has been linked with colorectal cancer, ulcerative colitis, and type 2 diabetes [18–20], while propionate has been linked to reduced weight gain, increased anorectic hormone secretion [21], and improved colonization resistance against enteric pathogens [22]. SCFA production varies strongly with microbiome composition; acetate and lactate are produced by many abundant microbes, whereas butyrate and propionate are synthesized by a more restricted subset [23]. Quantifying microbiome capacity to produce specific SCFAs is therefore essential for understanding how microbial activity shapes host metabolism and health.

Direct measurement of SCFAs remains difficult to interpret. Rapid host uptake renders fecal concentrations poor proxies for microbial production [4]. Moreover, spatiatemporal variability in microbial density, nutrient availability, and intestinal transit further limits localized measurements, which may not accurately reflect the total quantity of SCFAs released by the gut microbiota [1, 14]. We previously estimated SCFA production by integrating metagenomic abundances of known producers with in vitro secretion rates, revealing substantial inter-individual variation [4]. However, extrapolating strain phenotypes to complex microbiomes limits scalability and accuracy across heterogeneous datasets.

An alternative strategy infers fermentation pathway activity directly from metagenomic data independently of experimental characterization [24–27], assuming that gene abundance reflects metabolic output. However, this approach is challenged by multifunctionality of metabolic genes that participate in multiple pathways, ambiguity in public gene annotations, and arbitrary pathway detection thresholds that influence sensitivity.

Here, we systematically quantify the relative abundances of propionate- and butyrate-producing pathways across gut microbial species and human microbiomes. To address limitations of prior approaches [24–27], our pipeline defines required pathway genes based on experimentally validated model strains, filters ambiguous gene annotations, requires complete gene sets for pathway assignment, and cross-validates predictions against an extensive body of experimental literature.

Prior studies analyzed gene cascades to estimate pathway abundance [24, 27], and genome-resolved frameworks have inferred community-level SCFA production potential [28]. Building on these advances, we extend pathway-based inference in three key ways. First, we rigorously validate pathway predictions against reported fermentation phenotypes for >700 gut species. Second, we weight pathways by species relative abundance, enabling community-level estimates that reflect the functional impact of dominant taxa. Third, we examine conservation of fermentation pathways across taxonomic groups, revealing how abundance-weighted patterns give rise to emergent functional structure at higher taxonomic levels. Applied to large metagenomic datasets, this framework reveals substantial variation in butyrate and propionate pathway abundance associated with host age, early-life colonization, and inflammatory bowel disease (IBD).

## Methods and Materials

### Bioinformatics pipeline to estimate relative pathway abundance in metagenomic datasets

As outlined in Supplementary Fig. S1, we developed a five-stage pipeline to quantify butyrate- and propionate-producing pathways in bacterial genomes and metagenomic datasets.

#### Genome screening and pathway definition

Bacterial genomes containing butyrate- or propionate-producing pathways were identified from the Integrated Microbial Genomes (IMG) database [29] using Kyoto Encyclopedia of Genes and Genomes (KEGG) orthology annotations (release #1052023–01) [30] (Supplementary Table S1). Pathway requirements were defined using 37 experimentally characterized butyrate- and propionate-producing model strains spanning gut-associated and non-gut taxa [29, 30] (Supplementary Table S2). Genes consistently present in model strains were designated as essential for pathway presence. *EtfAB, acrC*, and *mmdD* were excluded due to inconsistent IMG annotations (**Supplementary Information**—**Sensitivity analysis of pathway gene requirements**). Entries for *bcd, but, ptb*, and *hbd* were further filtered using manually curated keyword criteria to remove non-specific hits (**Supplementary Information—Manual curation of unambiguous pathway gene descriptions**). Applying these requirements to 22 269 finished genomes identified 6152 butyrate- and 3237 propionate-positive genomes, with substantial variation among pathway prevalences (Supplementary Fig. S1(i)).

#### Profile HMM construction and strain classification

Profile hidden Markov models (HMMs) were generated for each pathway gene using HMMER v3.3.2 [31] and queried against >1000 genomes, including common gut strains [32] and experimentally validated producers [29, 30]. Hit sequences from queried strains returned by the HMMER search were filtered with a hit score cutoff of 50% of the lowest scoring model strain in accordance with previous studies [26, 27] and an observed score drop off.

To resolve strains encoding multiple pathways for the same fermentation product, we compared HMMER scores across all pathway-positive genes within each genome. Although genomic surveys indicate that multiple pathway variants can occur, they are relatively uncommon in gut bacteria [28], supporting the use of a single-pathway assumption in community-level estimates. Strains positive for multiple propionate or butyrate pathways were therefore assigned to the pathway with consistently higher scores relative to model strains (**Supplementary Information—Single-pathway determination for strains testing positive for multiple pathways**). However, 11 strains remained classified as multi-pathway carriers after this analysis, indicating that our approach captures genuine multi-pathway genomes while limiting misclassification.

Among 1109 queried gut strains passing score and gene completeness filters, 172 encoded propiogenic pathways and 189 encoded butyrogenic pathways. The most common pathways were SP for propionate and Ace for butyrate (Supplementary Fig. S1(ii)). Both butyrate- and propionate-producing pathways were found in 29 strains, including model strains *Megasphera elsdenii* and *Roseburia inulinivorans* known to have both pathways (Supplementary Table S2, S3).

#### Gene catalog construction and metagenomic mapping

Genes from pathway-positive genomes were compiled into a catalog containing >2000 nonredundant sequences (Supplementary Fig. S1(iii)). Metagenomic reads were mapped to this catalog using Bowtie2 v2.4.5 with the *—very-sensitive* preset in end-to-end mode [33] (Supplementary Fig. S1(iv)). Pathway counts were calculated from gene hit totals normalized by gene length and the number of genes in a pathway (**Methods and Materials—Pathway abundance count and normalization**).

#### Normalization and validation

To estimate the proportion of genomes encoding each pathway within a sample, pathway counts were normalized to the housekeeping gene *rplB* (Supplementary Fig. S1(v)), which encodes the 50S ribosomal protein and is nearly universal in bacteria (mean copy number ~ 1.03 in gut taxa) [27]. *RplB* counts were obtained by mapping reads against a catalog of *rplB* genes derived from a HMMER search requiring scores ≥400.

Some taxa may harbor multiple copies of *rplB* or duplicated SCFA pathway genes. However, *rplB* is widely used as a single-copy core marker for normalization in metagenomic studies, and its near-single-copy distribution minimizes bias when aggregating complex communities [27, 34–36]. Our pipeline further requires complete gene sets for pathway assignment, reducing miscounts due to paralogs. Validation against >700 experimentally characterized butyrate- and propionate-producing species shows that inferred pathway abundances recapitulate known fermentation phenotypes, supporting the robustness of our approach (**Results—Reported metabolic profiles validate pipeline**).

### Abundance data

To account for the varying abundances of different species, we utilized relative abundance values derived from metagenomics. To obtain these abundance numbers for a large pool of microbiota samples, we utilized a large collection of metagenomics data, incorporating data from 93 studies and ~18 000 samples [37]. The dataset contains donors with different health conditions and variations in diet. For the abundance calculations, we used for each species average abundance values across all samples in the collection.

### Pathway abundance count and normalization

For each metagenomic sample, the percentage of bacteria encoding a given pathway was calculated as *P_genomes_* = ∑((*h*_gene_ / *l_gene_*) / (*l_pathway_*)) / ∑(*h_housekeeping_* / *l_housekeeping_*). 100. Here, *h*_gene_ denotes the number of hits to a pathway gene, *l_gene_* is gene length, and *l_pathway_* is the number of genes in the pathway. *h_housekeeping_* and *l_housekeeping_* denote the hit count and sequence length of *rplB*, respectively. Normalization by *rplB* accounts for variability in sequencing depth across samples by scaling pathway gene counts to total genome equivalents within each metagenome, yielding an estimate of the fraction of genomes encoding a given pathway rather than absolute gene abundance.

Gene length is explicitly accounted for because longer genes are more likely to recruit reads due to their size, so normalization by *l_gene_* ensures that pathway abundance estimates reflect the number of genomes carrying the pathway rather than gene length biases. Normalization by the number of pathway genes (*l_pathway_*) further bases estimates on average per-gene evidence, preventing longer gene sets from being overrepresented. Assuming pathway genes are single copy in pathway-positive genomes, the estimator is independent of pathway length, enabling direct comparison across pathways.

For multi-pathway genes, *h*_gene_ was adjusted to reflect the proportion of single-pathway genes for each pathway in the same metagenomic sample under the assumption that the division of *h*_gene_ for multi-pathway genes is proportional to the pathway abundance, approximated by *h*_gene_ for single-pathway genes, calculated as follows: *h_gene_* = *h_o_* · (∑*h_s_* / ∑*h_m_*), with *h_o_* as the observed gene count of the multi-pathway gene, *h_s_* as the gene count for each single-pathway gene in the pathway of interest, and *h_m_* as the gene count for each single-pathway gene in all pathways. All boxplots shown are Tukey boxplots [38].

## Results

### Pathway abundance analysis

**Figure 1:**
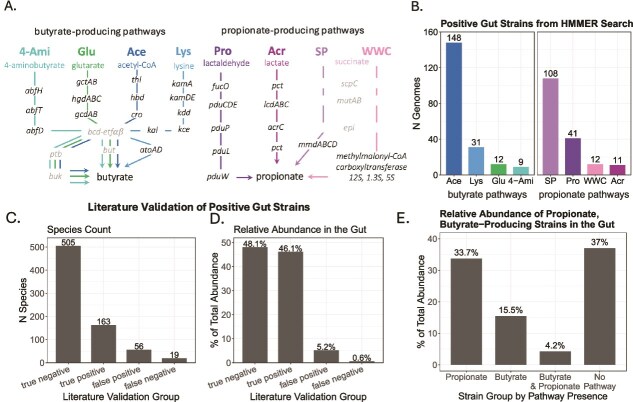
Quantification of butyrate and propionate pathway abundances. (A) Metabolic pathways for butyrate and propionate biosynthesis, named by their primary entry metabolite (colored labels; abbreviations in bold). Abbreviations: Glu, glutarate; Ace, acetyl-CoA; Lys, lysine; 4-Ami, 4-aminobutyrate/succinate; Pro, propanediol; Acr, acrylate; WWC, Wood–Werkman cycle; SP, sodium-pumping succinate pathway. Catalyzing genes are shown in black; gray indicates genes shared by multiple pathways. (B) Pathway presence in gut bacterial genomes predicted by the pipeline. (C and D) Validation of pathway predictions against fermentation product data reported in the literature for 743 species (11 species positive for multiple pathways excluded). (C) Species counts by prediction-validation category. (D) Relative gut microbiome abundance (percentage of total microbiota) by validation category. (E) Average abundance contributions of predicted butyrate and propionate producers versus strains with no detectable pathways. Bar labels denote corresponding proportions of total gut microbiota.

Eight well-characterized microbial fermentation pathways produce butyrate and propionate (Fig. 1A) [23, 39]. Pathways are referred to by their primary metabolite and consistently labeled throughout the text and figures. Butyrate pathways include glutarate (Glu), acetyl-CoA (Ace), lysine (Lys), and 4-aminobutyrate/succinate (4-Ami), while propionate pathways include propanediol (Pro), acrylate (Acr), and succinate, the latter subdivided into the Wood-Werkman cycle (WWC) and sodium-pumping pathway (SP).

To estimate pathway abundance, we extended the approach of Vital et al. [26, 27], constructing HMMs from experimentally characterized model strains (Supplementary Table S2) to predict pathway presence in >1000 human gut-associated genomes and metagenomes (Supplementary Fig. S1, **Methods and Materials**). About 20% of gut strains harbored butyrate pathways and 17% harbored propionate pathways. Ace and SP pathways dominated (Fig. 1B), consistent with prior reports that these are the principal routes for butyrate and propionate production in gut bacteria [23, 39–42].

### Reported metabolic profiles validate pipeline

To assess pipeline accuracy, we surveyed 318 studies and compiled reported fermentation phenotypes for 743 of the 1019 gut species analyzed (>70%, Supplementary Table S3). Although literature coverage is uneven, favoring well-studied taxa, this reflects current experimental knowledge rather than pipeline limitations, which operates at genome resolution under a rigorous, systematic framework.

Validation was performed at the species level, as most studies report species-level phenotypes, though strain-level variation may exist. Core enzymatic machinery for butyrate and propionate synthesis is broadly conserved within species, supporting species-level inference [41–44]. Comparison with reported phenotypes showed high concordance: 96% of species without detected pathways were true negatives, and 74% of species with predicted pathways were true positives, leaving 26% as false positives (Fig. 1C). This discrepancy may reflect limited experimental characterization available for these species, such as vitro conditions insufficient to induce SCFA production. Controlled fermentation studies show that environmental parameters can significantly alter fermentation product profiles even when the genetic capacity is present, suggesting that pathway presence may exceed observed production under certain experimental conditions [45–47].

Benchmarking against the existing pipeline of Vital et al. [26] for overlapping strains yielded >97% concordance, confirming strong agreement with established methods while maintaining conservative assignments to limit false positives (see **Supplementary Information – Benchmarking against existing pipelines**).

The abundance of species in the gut varies widely, with only a few species accounting for most of the biomass that constitutes the human gut microbiota [4]. To evaluate the ecological relevance of our predictions within the human gut microbiome, we weighted pathway predictions by species relative abundance in ~18 000 fecal metagenomes spanning diverse donors and health states [37] (**Methods and Materials**). Species with pathway predictions concordant with the literature comprised >94% of total gut microbial abundance, while discordant predictions represented <6% (Fig. 1D). Predicted butyrate- and propionate-producers together accounted for ~53% of total microbial abundance (Fig. 1E), emphasizing the importance of these pathways in the human gut. Together, these results validate our pipeline as a robust tool for inferring butyrate and propionate production potential from metagenomic data, particularly for the dominant species that drive community-level fermentation.

### Taxonomic distribution of butyrate and propionate producers

To assess the phylogenetic diversity of predicted butyrate and propionate producers and the validity of using single strains as taxonomic representatives, we analyzed the taxonomic distribution of pathway-positive genomes. Assignments were made at the genome level based on complete metabolic pathways (see **Methods and Materials—Bioinformatics pipeline to estimate relative pathway abundance in metagenomic datasets**), and taxonomic labels were used only to summarize and aggregate these predictions, not to infer metabolic function a priori.

**Figure 2:**
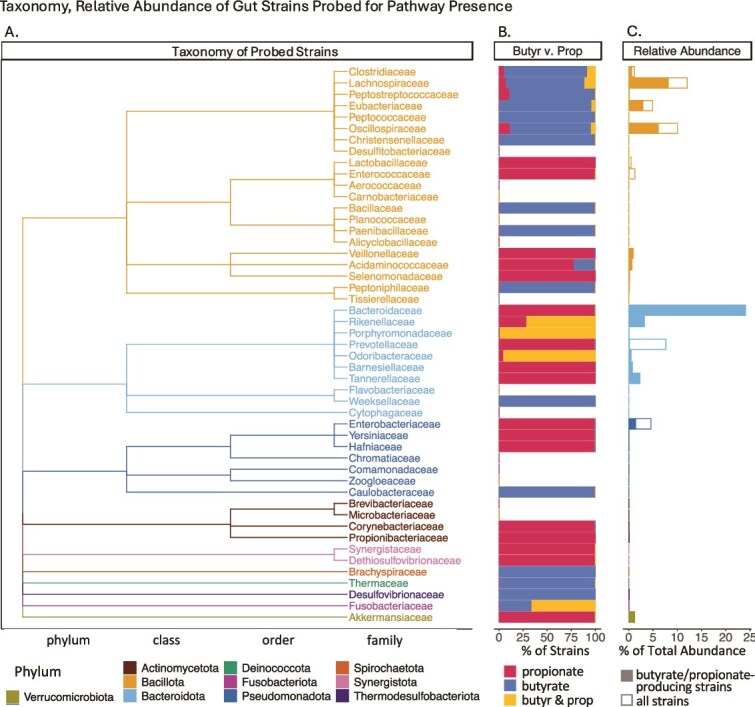
Grouping of predicted butyrate and propionate producers and relative abundance in the gut at the family level. (A) Phylogenetic tree of 16S rRNA of families probed for pathways. Taxonomic branch points are indicated beneath the tree, with branches color-coded by phylum. (B) Proportion of predicted butyrate- and propionate-producing strains in each family. (C) Percentage of the total gut microbial abundance of each family in the gut microbiome as average relative abundance. Color-filled portions of each bar represent the abundance of predicted butyrate or propionate producers.

Among 1019 gut species [29, 30, 32], predicted producers spanned 36 families and 10 phyla, reflecting broad phylogenetic distribution (Fig. 2A). Notably, within the families, predicted producers often—but not always—produced the same SCFA (Fig. 2B, red and blue bars). For example, all Bacteroidaceae strains were predicted propionate producers, while most Lachnospiraceae strains produced butyrate. However, at the phylum level, this separation is less accurate: Bacteroidota contains both propionate- and butyrate-producing strains, including Weeksellaceae, which produce only butyrate. This emphasizes the necessity of taxonomic analysis at the family-level or finer for accurately estimating fermentation product secretion from microbiome composition.

Weighting predictions by species abundance in the gut clarifies patterns of classifying butyrate and propionate producers by taxonomy. Bacillota and Bacteroidota are the most abundant phyla, with dominant families—Lachnospiraceae, Eubacteriaceae, and Oscillospiraceae in Bacillota, and Bacteroidaceae and Prevotellaceae in Bacteroidota—accounting for most biomass (>5% relative abundance) (Fig. 2A, C). Outside of these phyla, only the Enterobacteriaceae family reaches ~5% abundance.

Within these dominant families, predicted butyrate- and/or propionate-producing strains account for >40% of the relative abundance in the gut. Well-described genera that do not produce butyrate or propionate (e.g. *Blautia* and *Dorea* represented in the non-producing fraction of Lachnospiraceae, Fig. 2C) in these same families constitute a substantially smaller cumulative relative abundance in the gut (<18%, Fig. 2C). Overall, most Bacillota abundance is butyrate-producing and most Bacteroidota abundance is propionate-producing (Fig. 2B). Thus, while taxonomic classification alone is insufficient to determine SCFA production for individual taxa, the strong abundance-weighted enrichment of butyrate-producing pathways within dominant Bacillota and propionate-producing pathways within dominant Bacteroidota suggests that, at the level of highly prevalent gut phyla, phylum-level composition can serve as a coarse but informative approximation of community-wide SCFA secretion potential.

### Variation of pathway abundance in healthy humans

Building upon these taxonomic insights, we next sought to directly compare the relative abundances of butyrate versus propionate producers across metagenomic samples. Pathway abundance was estimated for each sample (Supplementary Fig. S1(v)) and normalized using length-adjusted *rplB* counts to account for sequencing depth, yielding the fraction of genomes encoding each pathway (**Methods and Materials—Pathway abundance count and normalization**).

**Figure 3:**
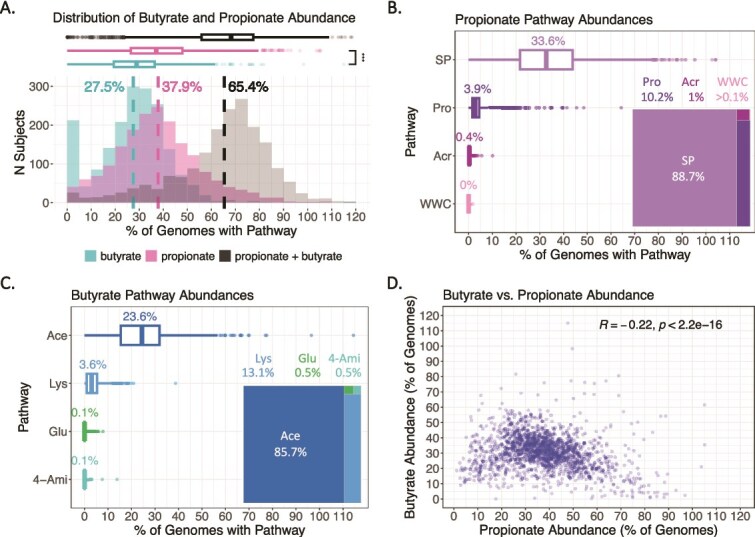
Variation of propionate and butyrate pathway abundance in healthy individuals. (A) Distribution of the percentage of total bacteria containing butyrate pathways, propionate pathways, and the total combined pathway abundance. The average fraction of bacteria containing propionate pathways is significantly higher than that of butyrate pathways. Dashed lines represent the means. (B and C) Abundance of the different propionate and butyrate pathways. Numbers above the box plots indicate means. Insets show the relative abundances of individual butyrate/propionate pathways in comparison to the total pathway abundance. Data from 2317 metagenomic samples from *n* = 1831 healthy individuals [48–56]. Significance in panel A between butyrate and propionate pathway abundances was confirmed by the Games-Howell test (*P* < .05). In panels B and C, significance was confirmed by the Kruskal-Wallis test, with post-hoc Games-Howell testing for SP vs. Pro pathway abundances in B, and Ace vs. Lys pathway abundances in C (*P* < .05). (D) Correlation between propionate and butyrate pathway abundances, with Spearman’s rank correlation coefficient shown. Data from 2115 metagenomic samples from *n* = 1629 healthy individuals over 6 months old [48–56].. The small portion of individuals with pathway abundances exceeding 100% (A, D) may represent cases where strains harbor multiple copies of pathway genes or contain more than one pathway.

We analyzed raw reads from >1500 healthy individuals of diverse ages and geographical regions (**Supplementary Information—Metagenomic Datasets Included in this Study**) [48–56], allowing us to examine the natural variation of SCFA pathway prevalence in a wide population. Overall, both butyrate and propionate pathways were highly abundant across individuals, with an average of 65% of bacteria in the gut carrying at least one butyrate or propionate pathway (Fig. 3A). Propionate pathways were more abundant (38% on average) than butyrate, with <1% of samples showing <1% propionate abundance (Fig. 3A, pink distribution). Among propionate pathways, SP dominated, followed by Pro; the others were rare (Fig. 3B).

Butyrate pathways averaged 28% relative abundance and showed greater inter-individual variation, consistent with previous reports [26, 27], with ~8% of samples exhibiting negligible levels (Fig. 3A, teal distribution). The Ace pathway contributed most to butyrate abundance, as previously described [26, 27], followed by Lys (Fig. 3C). We observed a weak anticorrelation between butyrate and propionate pathway abundance (Fig. 3D), aligned with the expectation that gut bacteria typically harbor only one of the eight butyrate and propionate pathways. These results underscore the complementary roles of butyrate and propionate production in promoting anaerobic growth in the gut microbiome.

### Variation with age

To investigate host factors contributing to pathway abundance variation, we first examined age, an established key factor in shaping gut microbiome composition [48, 57]. Subjects were grouped into six age categories biologically relevant to distinct gut microbiota compositions [57], and pathway abundance was compared across groups.

**Figure 4:**
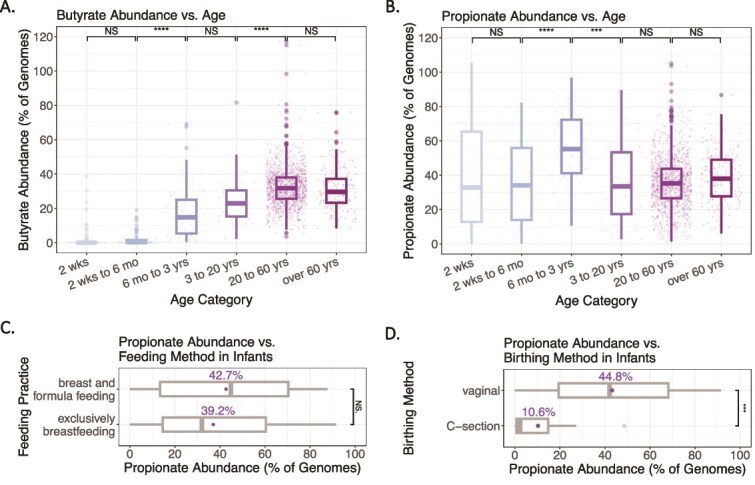
Variation in butyrate and propionate pathway abundances by age. (A and B) Abundance of butyrate and propionate pathways across different age categories. Data from 2124 metagenomic samples from N = 1702 healthy individuals with available data on age [48–56]. (C and D) Propionate pathway abundance in infants less than 2 weeks old by feeding method and birthing method. Numbers above the box plots represent mean values. Data from N = 78 (C) and N = 90 (D) infants with available data on feeding method and birthing method [50]. Significance in A and B determined by the Kruskal-Wallis test and in A, B, C, and D by the Games-Howell test (*P* < .05).

Butyrate pathways were rare in infants but increased sharply after six months, consistent with previous work [26, 27, 58], continuing to rise with age and peaking in adults over 20 years at 32.1% average abundance (Fig. 4A). The Ace pathway was abundant in children 6 months–3 years, while Lys abundance rose later, from 3 to 20 years (Supplementary Fig. S2A).

Propionate pathway abundance exhibited a different pattern. Infants displayed high propionate pathway abundance with considerable variation (Fig. 4B). This variation stabilized somewhat with age, peaking at 56.5% in children from 6 months to 3 years. SP and Pro pathways remained the most prevalent across all ages (Supplementary Fig. S2B).

Given the marked variation of propionate pathway abundance in infants, we further investigated the influence of feeding practices and birthing methods in infants under 2 weeks old. No significant difference in propionate pathway abundance was observed between exclusively breastfed infants and those fed a combination of breast milk and formula (Fig. 4C), but birthing method had a strong effect. Vaginally born infants averaged 44.8% propionate pathway abundance, while Cesarean-born infants averaged substantially lower at 10.6% (Fig. 4D).

### Variation with intestinal health

**Figure 5:**
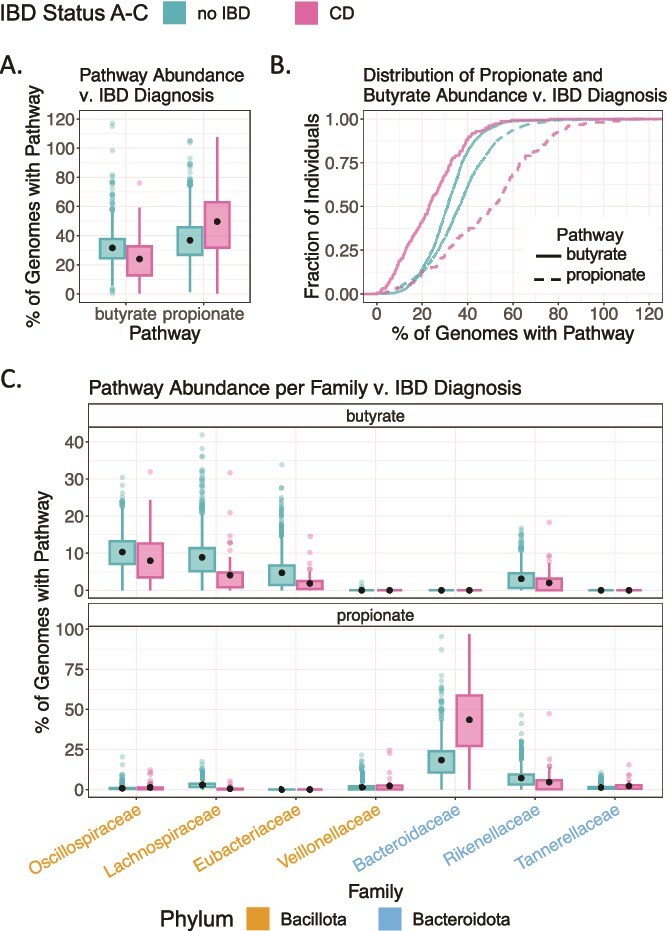
Variation in propionate and butyrate abundance in healthy versus IBD-diagnosed individuals. (A and B) Opposite trends in the variation of butyrate and propionate abundances in healthy subjects versus individuals with IBD. Significance for mean butyrate and propionate pathway abundances between IBD and healthy groups confirmed by Kruskal-Wallis test and Games-Howell test (*P* < .05). (C) Variation in propionate and butyrate abundance across the most abundant bacterial families (relative abundance >5%), colored by phylum (ochre for Bacillota, blue for Bacteroidota). All plots are colored by IBD status (teal for no IBD; pink for IBD). Black dots on box plots indicate means. Data from 3733 samples from *N* = 1810 subjects over the age of 3 with available data on IBD diagnosis [48–56].

To explore the relationship between gut health and pathway abundance, we compared healthy individuals with those diagnosed with Inflammatory Bowel Disease (IBD), including Crohn’s Disease (CD) and Ulcerative Colitis (UC). We observed distinct patterns in the abundance of propionate and butyrate pathways based on IBD status. Butyrate pathway abundance was 8% higher in healthy subjects than in CD patients (Games-Howell test, *P* < 6.72 · 10^−10^, Fig. 5A), consistent with previous findings [27], but did not differ significantly between healthy and UC individuals. In contrast, propionate pathway abundance exhibited the opposite trend and was 12.8% lower in healthy subjects compared to CD patients (Games-Howell test, *P* = 4.93 · 10^−10^, Fig. 5A). In healthy individuals, butyrate and propionate abundances were similar (5.2% difference, Fig. 5B, teal lines). Conversely, in individuals with CD, propionate pathway abundance exceeded butyrate by 26.5% (Fig. 5B, pink lines).

These differences in butyrate and propionate relative abundance between IBD and non-IBD subjects were driven by shifts in dominant bacterial families within Bacillota and Bacteroidota (Fig. 5C). Higher butyrate abundance in healthy subjects reflected increases in Bacillota families Oscillospiraceae, Lachnospiraceae, and Eubacteriaceae, while higher propionate abundance in IBD was largely explained by an increase in the Bacteroidota family Bacteroidaceae. No significant changes were observed in the abundance of *Escherichia coli* or other propionate-producing members of Pseudomonadota. Together, these results indicate that disease-associated shifts in SCFA pathways are mediated by family-level changes strongly structured by phylum.

## Discussion

The continuous exchange of fermentation products between the gut microbiota and the host represents a central metabolic interface shaping host physiology. In the anaerobic environment of the large intestine, microbial growth is sustained primarily through carbohydrate fermentation, leading to the production of SCFAs that influence immune function, epithelial health, and systemic metabolism [3]. Because individual SCFAs exert distinct physiological effects, understanding how the capacity to produce specific fermentation products is distributed across gut microbial communities is critical for interpreting microbiome–host interactions in health and disease.

In this study, we adapted a previously-introduced genome-resolved bioinformatics framework [26, 27] to estimate the relative abundance of butyrate- and propionate-producing fermentation pathways in human gut microbiomes. By curating complete pathway gene sets from experimentally characterized model strains, filtering non-specific gene annotations, requiring the presence of complete gene cascades for pathway assignment, and weighting pathway predictions by species abundance, our approach advances beyond earlier taxonomic or marker-based methods for inferring SCFA production potential. Importantly, we cross-validated pathway assignments against an extensive experimental literature spanning over 700 species, providing confidence that predicted pathway presence reflects known fermentation phenotypes. This validation step distinguishes our framework from prior purely computational approaches and enables scalable inference across large and heterogeneous metagenomic datasets.

Our taxonomic analysis revealed that the capacity to produce butyrate or propionate is phylogenetically structured but heterogeneous within taxonomic groups. While pathway presence is often conserved within families, consistent with earlier genome-resolved analyses [3, 28], substantial variation exists within families and across phyla, underscoring the limitations of assigning fermentation potential based solely on taxonomy. Nevertheless, when weighted by species abundance, dominant contributors to the adult gut microbiome exhibit strong enrichment of butyrate-producing pathways within Bacillota and propionate-producing pathways within Bacteroidota. This abundance-weighted analysis reveals that dominant taxa largely determine community-wide SCFA production capability, supporting the use of coarse taxonomic summaries to capture broad butyrate- and propionate-producing functional trends at the community level.

Applying this pipeline across more than 1500 individuals, we observed pronounced inter-individual variation in pathway abundances, with approximately two-thirds of gut bacteria encoding at least one butyrate- or propionate-producing pathway. Across adult samples, propionate-producing pathways were, on average, more abundant than butyrate-producing pathways. The abundance of the two pathway classes were anticorrelated, and this inverse relationship suggests ecological competition between butyrate- and propionate-producing microbes, perhaps reflecting overlap in substrate utilization during carbohydrate and amino acid fermentation. Such trade-offs are consistent with known metabolic constraints in anaerobic ecosystems, where shifts in substrate availability or redox balance can favor alternative fermentation end products [3, 24, 59].

Pathway abundances varied markedly across age groups, revealing developmental patterns in gut fermentative capacity. Infants exhibited consistently low abundance of butyrate-producing pathways but relatively high abundance of propionate-producing pathways. This observation aligns with established models of early-life microbiome development, in which facultative anaerobes and *Bacteroides* species—predicted propionate producers by our analysis—dominate prior to the maturation of obligate anaerobic communities enriched in butyrate producers [50, 60]. We further observed a strong association between propionate pathway abundance and birthing method, with vaginally delivered infants harboring higher propionate pathway abundance than infants delivered by Cesarean section. Vaginal delivery is known to facilitate early colonization by maternal gut microbes, primarily *Bacteroides* and other Bacteroidetes members that largely encode for propionate production, whereas Cesarean delivery is associated with delayed acquisition of these taxa [61, 62]. These results suggest that differences in early microbial colonization associated with birth mode translate into measurable differences in fermentative potential, highlighting how early-life exposures may shape metabolic interactions between the microbiome and the host.

Consistent with previous studies, we found that butyrate pathway abundance was significantly reduced in individuals with CD compared to those without, whereas propionate pathway abundance exhibited the opposite trend. While propionate production itself has not been directly implicated as detrimental to gut health, a reduction in butyrate-producing populations may contribute to altered resource (e.g. host-derived nutrients) availability that facilitates the expansion of propionate-producing populations, as reflected in our observed increase of *Bacteroides* in CD-afflicted individuals. Given the central role of butyrate in maintaining epithelial integrity and modulating inflammation [3, 29], such shifts in fermentative balance may contribute to disease-associated dysbiosis. The expansion of propionate-producers may also be influenced by inflammation-driven factors such as oxidative stress tolerance or altered lipopolysaccharide structures that selectively favor propionate producers independently of changes in the abundance of butyrate producers. Longitudinal studies integrating pathway abundance with disease progression and treatment response will be necessary to elucidate mechanisms for inflammation-associated shifts in SCFA-producing community composition.

Several limitations of this sequence-based approach should be acknowledged. The presence of a complete fermentation pathway indicates metabolic potential but does not directly measure pathway activity or flux. Regulation of fermentation product formation depends on environmental factors such as substrate availability, pH, redox state, and microbial interactions, which are not captured by genomic data alone. Moreover, some organisms encode multiple alternative fermentation pathways, and relative flux through these pathways may vary dynamically in response to ecological context. As a result, estimated pathway abundances should be interpreted as proxies for community-level fermentative capacity rather than direct measures of SCFA production. Integrating this framework with metatranscriptomic, metabolomic, or isotope-tracing approaches will be an important direction for future work.

Building on this framework, future studies can explore how gut microbiome fermentative capacity interacts with host physiology, behavior, and diet. Applying this pipeline to link butyrate and propionate pathway abundances to dietary intake or specific nutrient availability could provide mechanistic insight into diet–microbiome–host interactions. Integrating pathway-based predictions with complementary data types—such as metatranscriptomics, metabolomics, or isotope tracing—will further enable assessment of actual pathway activity and flux, overcoming limitations of sequence-based inference. Beyond butyrate and propionate, this framework that combines genome-resolved pathway mapping, literature benchmarking, and abundance-weighted analysis represents a methodological advance that can be applied to other fermentation pathways, such as those producing branched-chain fatty acids or other metabolites with potential health implications [63, 64]. Overall, this scalable, validated, and abundance-weighted approach provides a robust platform for dissecting microbial metabolic potential across large, heterogeneous cohorts and lays the foundation for future studies linking microbiome function to host health.

## Supplementary Material

ycag075_Supplemental_Files

## Data Availability

The pipeline for running the HMMER search, filtering the HMMER, constructing the gene catalog, performing the BOWTIE2 read-mapping, and calculating pathway abundances is available at https://github.com/rchristensen26/Butyrate_Propionate_Comparative_Analysis. Included in the pipelines are all input files, accession numbers for metagenomic data analyzed, and data output files.
